# IDENTIFYING GAPS AND MISCONCEPTIONS AND EXAMINING PREDICTORS OF ALZHEIMER’S DISEASE KNOWLEDGE IN KOREAN AMERICANS

**DOI:** 10.1093/geroni/igac059.1877

**Published:** 2022-12-20

**Authors:** Michin Hong, Sang Lee

**Affiliations:** Indiana University, Indianapolis, Indiana, United States; San Jose State University, San Jose, California, United States

## Abstract

This study examined Alzheimer’s disease (AD) knowledge and its predictors among Korean Americans (KAs). A total of 268 KAs in the Greater Washington metropolitan area participated in the study and completed a cross-sectional survey. Using the Alzheimer’s Disease Knowledge Scale (ADKS), overall and domain knowledge was assessed. Multiple regression analyses were conducted with predictors including exposure to AD, social engagement, sources and frequency of health-related information, stigmatic beliefs (pity, antipathy, and social distance), English proficiency, and education. KAs reported 59% accuracy in overall AD knowledge. They were most knowledgeable about assessment and diagnosis domain and least knowledgeable about caregiving domain. Regression analyses showed that having more education is associated with greater overall and certain domain knowledge. Having more pity stigmatic beliefs is related to greater knowledge in both life impact and caregiving domains while having less pity stigmatic beliefs is associated with more risk factor knowledge; having less social distance stigmatic beliefs is associated with greater life impact knowledge; and having less antipathy stigmatic beliefs is related to better caregiving knowledge. Our findings revealed areas of misconceptions and knowledge gaps in KAs which need to be addressed in educational interventions. Different knowledge status across the domains demonstrates a multi-dimensional nature of AD knowledge. Multivariate findings confirmed the robust role of education in AD knowledge. Effect of different AD stigmatic beliefs on certain AD knowledge domains suggests ways of how stigma change can be efficient for the purpose of increasing AD domain knowledge in KAs.

